# Integrated metagenomic and 16S rRNA analysis reveals temporal associations between resistance genes and microbial communities during dairy manure composting

**DOI:** 10.1038/s41598-026-37092-y

**Published:** 2026-02-05

**Authors:** Yuan Zhou, Kaiyue Liu, Ping Gong, Jian Wu, Zhuqing Ren, Erguang Jin

**Affiliations:** 1 Huazhong Agricultural University, Wuhan, Hubei China; 2 Wuhan Academy of Agriculture Sciences, Wuhan, Hubei China

**Keywords:** Antibiotic resistance genes (ARGs), Dairy manure composting, Horizontal gene transfer, Microbial community succession, Resistome co-selection, Computational biology and bioinformatics, Environmental sciences, Microbiology

## Abstract

Dairy manure composting is widely applied to stabilize organic waste and reduce environmental pollution, yet the behavior of resistance determinants during this process remains insufficiently resolved. In this study, shotgun metagenomic sequencing was used to characterize temporal changes in antibiotic resistance genes (ARGs), metal resistance genes (MRGs), biocide resistance genes (BRGs), mobile genetic elements (MGEs), and microbial community composition during dairy manure composting. Rather than inferring direct mechanistic causation, our analyses focused on identifying statistically supported trends, associations, and co-occurrence patterns across composting stages. We observed a rapid decline in the relative abundance of ARGs compared with MRGs and BRGs during the thermophilic phase, coinciding with increasing temperature, while specific genes such as sul2 persisted throughout the process. Shifts in microbial community composition, particularly changes in the relative dominance of *Actinobacteria* and *Proteobacteria*, were significantly associated with variations in resistome profiles. Correlation and network analyses further revealed strong associations among ARGs, MRGs, BRGs, and MGEs, suggesting potential co-selection and horizontal gene transfer linkages without implying direct causal mechanisms. In addition, several opportunistic bacterial genera showed positive associations with aminoglycoside- and macrolide–lincosamide–streptogramin-type ARGs, indicating possible dissemination risks following compost application. Overall, this study provides an integrated, association-based overview of resistome and microbial community dynamics during dairy manure composting and highlights the importance of considering multiple resistance determinants when evaluating composting as a manure management strategy.

## Introduction

Antimicrobial resistance (AMR) is a critical global public health challenge with profound implications for human, animal, and environmental health, as recognized by the World Health Organization and other international agencies^1^ While much focus has been placed on clinical settings, increasing evidence suggests that environmental reservoirs of resistance, particularly those associated with agricultural practices, contribute significantly to the spread of antibiotic resistance genes (ARGs) in natural ecosystems^2,3^. Among agricultural sources, livestock manure has emerged as a critical reservoir and vector for ARG dissemination due to the routine use of antibiotics for growth promotion and disease prevention in intensive farming systems^4–6^. Dairy farming plays a central role in global agriculture and food security; however, its intensification has been accompanied by increased use of veterinary antibiotics, heavy metals (e.g., copper and zinc as feed additives), and disinfectants. These compounds exert selective pressure on microbial populations, promoting the enrichment and persistence of ARGs, metal resistance genes (MRGs), and biocide resistance genes (BRGs)^7,8^. Resistance genes are often harbored on mobile genetic elements such as plasmids, transposons, and integrons, facilitating their horizontal transfer across bacterial species, including potential human pathogens^9^.

Manure from dairy farms is frequently recycled as organic fertilizer to enhance soil fertility and crop productivity. However, this practice can inadvertently introduce high loads of ARGs, pathogenic microorganisms, and residual antimicrobials into the environment^10,11^. When applied to agricultural fields, untreated or poorly managed manure can contaminate soil, water, and food chains with resistant bacteria, posing risks to animal and human health. Composting is therefore widely applied as a manure management strategy to stabilize organic waste, reduce pathogens, and mitigate resistance-related environmental risks^12,13^. Composting involves a succession of microbial-mediated aerobic degradation processes, typically progressing through mesophilic, thermophilic, and maturation phases, characterized by shifts in temperature, oxygen availability, moisture, and nutrient dynamics^14^. During the thermophilic phase, temperatures can reach 55–70 °C, which is generally effective at inactivating many pathogenic microorganisms and extracellular DNA. However, the efficacy of composting in reducing ARGs and co-resistance mechanisms remains variable and often incomplete^15^. Recent studies indicate that although overall ARG abundance often declines during composting, specific resistance genes can persist or re-emerge, reflecting selective pressures associated with microbial succession and environmental stressors^16^. Throughout this study, references to “pathogenic” or “opportunistic” taxa are based on their documented clinical relevance in human or animal health contexts. It is acknowledged that many of these microorganisms are ubiquitous in environmental systems and do not inherently pose a health risk outside susceptible hosts or specific exposure scenarios. Their identification in composting systems is therefore interpreted in an ecological and association-based context rather than as evidence of active pathogenicity.

In addition, certain MRGs and BRGs tend to be more stable than ARGs under composting conditions due to their association with essential cellular functions and resistance to thermal degradation^17^. This persistence is particularly concerning because metals and biocides can indirectly maintain or enrich ARGs through genetic linkage, cross-resistance, and shared regulatory mechanisms, underscoring the importance of investigating resistance determinants as interconnected networks rather than isolated traits^7,9^. Microbial community succession is a key driver of resistance gene dynamics during composting, as shifts in dominant taxa directly influence resistance gene retention and host availability. These microbial shifts affect not only biodegradation efficiency but also the resistome, the collective pool of resistance genes within the community. For example, thermophilic genera such as Bacillus, Geobacillus, and Thermus may favor the maintenance of specific ARGs, particularly when resistance genes are carried on thermostable plasmids^18–20^.

To accurately capture resistance dynamics, resistance gene profiles must be evaluated alongside microbial community composition and mobile genetic elements. Targeting ARGs together with MRGs and BRGs is essential because these gene classes frequently co-occur and collectively shape resistance persistence through co-selection and horizontal gene transfer mechanisms. Despite these advances, significant knowledge gaps remain regarding how microbial succession mediates pathogen survival and resistance gene co-selection across composting stages. Addressing these gaps is essential for developing effective manure management strategies that mitigate AMR risks in agricultural systems. We hypothesized that dairy manure composting would differentially influence the dynamics of resistance determinants, with antibiotic resistance genes (ARGs) declining more rapidly than metal resistance genes (MRGs) and biocide resistance genes (BRGs) due to temperature-driven microbial succession. We further hypothesized that shifts in microbial community composition, together with the persistence of mobile genetic elements (MGEs), would be associated with the co-occurrence and potential co-selection of ARGs, MRGs, and BRGs across composting stages, resulting in the persistence of specific resistance genes despite overall resistome attenuation. This study therefore focuses on the persistence and selective enrichment of ARGs, MRGs, and BRGs during dairy manure composting. While most ARGs decline with increasing temperature, specific genes such as sul2 demonstrate resilience, suggesting strong selective pressures^21,22^. By integrating resistance gene profiling with microbial community analysis and mobile genetic element associations, this study provides a mechanistic understanding of resistance gene co-selection during composting.

## Materials and methods

### Composting and sampling

Fresh cow dung and sawdust were sourced from the experimental dairy farm associated with the Institute of Animal Husbandry and Veterinary Medicine, Wuhan Academy of Agricultural Sciences. Bedding materials utilized in broiler breeding were acquired from local rural farmers. The composting experiment was carried out at the dairy farm of the Animal Husbandry and Veterinary Institute, Wuhan Academy of Agricultural Sciences, spanning a duration of 35 days. The composting setup involved a plastic container, dimensions 1060 mm by 950 mm by 765 mm, insulated with thermal wrapping materials. Fresh cow dung, broiler breeding bedding, and sawdust were combined in a ratio of 12:3:5, thoroughly mixed, with the total mass of the mixture amounting to 200 kg. Microbial agents in powdered form, comprising bacillus, faecalis, lactic acid bacteria, yeast, etc., with a total bacterial count of ≥ 1.0 × 10^10 CFU·g^-1 (Wuhan XuRun Environmental Protection Technology Co., Ltd.), were added to the mixture before being placed into the composting container. Prior to initiating the experiment, the carbon-to-nitrogen (C/N) ratio of the composting materials was adjusted to 25:1, and the initial moisture content was set to 58.26% (wet weight basis, w/w). Throughout the composting period, the piles were manually turned on days 7, 14, 20, 25, and 30 to ensure adequate aeration. Moisture content was measured daily using a gravimetric method. Based on these measurements, deionized water was added on day 7 to adjust the moisture content to 60% (w/w). Compost samples were collected on days 0, 3, 10, 20, and 35 from the upper, middle, and lower layers (10, 30, and 50 cm from the surface) using a five-point sampling strategy. After thorough homogenization, approximately 500 g of each composite sample was obtained using the quartering method and stored in sterile, sealed plastic bags for subsequent analyses. The composite samples were divided into two subsamples. One subsample was stored at 4 °C for subsequent determination of physicochemical properties, while the other subsample was immediately frozen at − 80 °C for DNA extraction and metagenomic analysis. All experiments were conducted in triplicate using a randomized design to minimize bias and experimental error.

### DNA extraction and metagenomic sequencing

DNA from samples was extracted using the DN Easy PowerSoil Kit, and the extracted genomic DNA was evaluated via 0.7% agarose gel electrophoresis. The DNA concentration was precisely quantified using a Qubit 4.0 Fluorometer (Life Technologies, Carlsbad, CA, USA). Shotgun metagenomic sequencing was employed in this study; no targeted amplicon sequencing was performed, and therefore no hypervariable regions or PCR primers were used. Libraries were constructed using the VAHTS Universal Plus DNA Library Prep Kit for MGI V2, which involves fragmenting the input DNA, end-repairing the fragmented DNA to create blunt ends, phosphorylating the 5’ ends, adding an adenine (dA) to the 3’ ends, and finally ligating adapters to the product’s ends. For libraries with attached adapters, fragment size selection was performed using two rounds of VAHTS DNA Clean Beads (0.56×, 0.2×). This was followed by PCR amplification of the original library using a high-fidelity polymerase to ensure an adequate total library quantity. The concentration of each library was precisely measured using a Qubit 4.0. Sequencing was conducted on the MGI T7 platform with PE150. The PE150 read configuration was selected because it provides an effective balance between sequencing depth, read length, and assembly accuracy for complex environmental metagenomes, improving taxonomic assignment, functional gene annotation, and detection of resistance genes and mobile genetic elements. The PE150 strategy is widely applied in environmental shotgun metagenomics and has been shown to provide sufficient resolution for resistome profiling, reliable gene annotation, and accurate taxonomic classification while maintaining high sequencing throughput and cost efficiency^3,23,24^. The metagenomic data was deposited to National Centre for Biotechnology Information under Accession: PRJNA1301025 and ID: 1,301,025.

### ARG, MRG and species annotation

For the annotation of ARGs, clean reads obtained from metagenomic sequencing were directly annotated using the ARGs-OAP v2.0 tool, referencing the SARG database^23^. Additionally, metal resistance genes and biocide resistance genes were annotated by referencing the BacMet database^7^. Species taxonomic annotations were performed using Kraken2 with default parameters on the clean reads, classifying them at various taxonomic levels including kingdom, phylum, class, order, family, genus, and species. This process involved tallying the number of reads annotated to different microbial species in each sample, resulting in a species annotation abundance table that detailed the distribution of microbial species across the samples.

### Data analysis and visualization

With all the experiments run in triplicate and randomized, all statistical analyses and visualization were performed using R version 4.0.2 (https://www.R-project.org/ )^25^. Data manipulation was primarily conducted using the dplyr package (https://CRAN.R-project.org/package=dplyr)^26^, while graphical outputs were generated using ggplot2^27^. Heatmaps illustrating the abundance of resistance genes were constructed using the P-heatmap package^28^, focusing on the top 30 genes ranked by mean abundance across all samples. Network analyses were based on Pearson correlation coefficients with Benjamini–Hochberg (BH) correction for multiple testing, implemented using the linkET package^29^. Prior to analysis, genes or species detected in fewer than 60% of samples were excluded, followed by abundance-based filtering to reduce false positives. Thresholds were set at > 0.005 for 16S rRNA genes, > 0.02 for cellular genes, > 5 ppm for resistance genes, and > 0.5% relative abundance for species across samples. Significant correlations (|r| > 0.8, *p* < 0.05) were visualized as network diagrams using the ggraph package^30^. Variance partitioning analysis was performed using the vegan package^31^. Differences in resistance gene abundance, microbial community composition, and other parameters across composting stages were assessed using non-parametric Kruskal–Wallis tests followed by Dunn’s post-hoc test with BH correction. When assumptions of normality and homogeneity of variance were met, one-way analysis of variance (ANOVA) was applied, followed by Tukey’s HSD test for pairwise comparisons. Statistical significance was defined at *p* < 0.05 unless otherwise stated.

### Quality control

Quality control and bioinformatic processing were rigorously implemented for shotgun metagenomic datasets. Raw reads were quality-filtered using standard thresholds for base quality scores and read length, and host-derived sequences were removed prior to downstream analyses. For metagenomic data, resistance genes were identified using a direct read-based annotation approach against curated databases with clearly specified version numbers, minimum sequence identity, coverage, and read-count thresholds to ensure reliable ARG, MRG, and BRG detection. For 16S rRNA amplicon data, chimera detection and removal were performed, followed by rarefaction and normalization to account for differences in sequencing depth across samples. Sequencing depth per sample and database versions used for taxonomic and resistance gene annotation are now explicitly reported in the revised Methods section, ensuring transparency, reproducibility, and robustness of the analytical workflow.

## Results

### Composting process

**Fig. 1:**
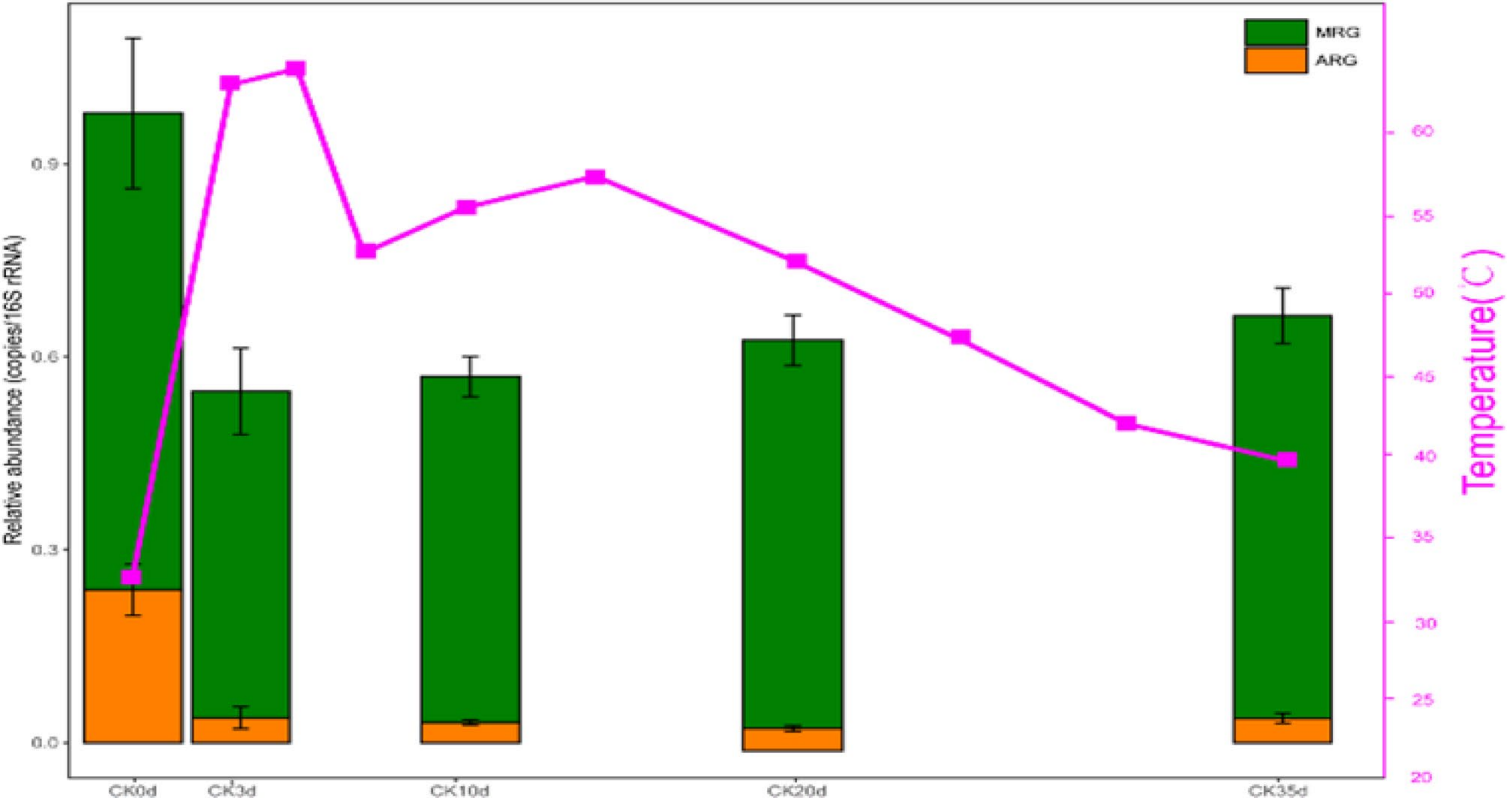
Composting process temperature variations and their impact on the total relative abundance of ARGs and MRGs after a given time (Ck0d is 0 days, Ck3d is 3 days, Ck10d is 10 days, Ck20d is 20 days and Ck35d is 35 days).

During the composting period of the manure, there was observed rapid increase in the pile’s temperature, rising from 32 °C on day 0 to a peak of 63 °C by day 3. It then declined to 52.5 °C by day 7 and steadily decreased to 39.5 °C by day 35. A slight increase was observed between day 7 and day 14, with the temperature of the reactor body marginally peaking at 57.3 °C. Furthermore, differences in the relative abundance of ARGs, BRGs and MRGs, with distinct trends evident in their temporal profiles was observed (Fig. 1).

### Dynamics of ARGs, MRGs and BRGs during composting

#### ARGs

**Fig. 2:**
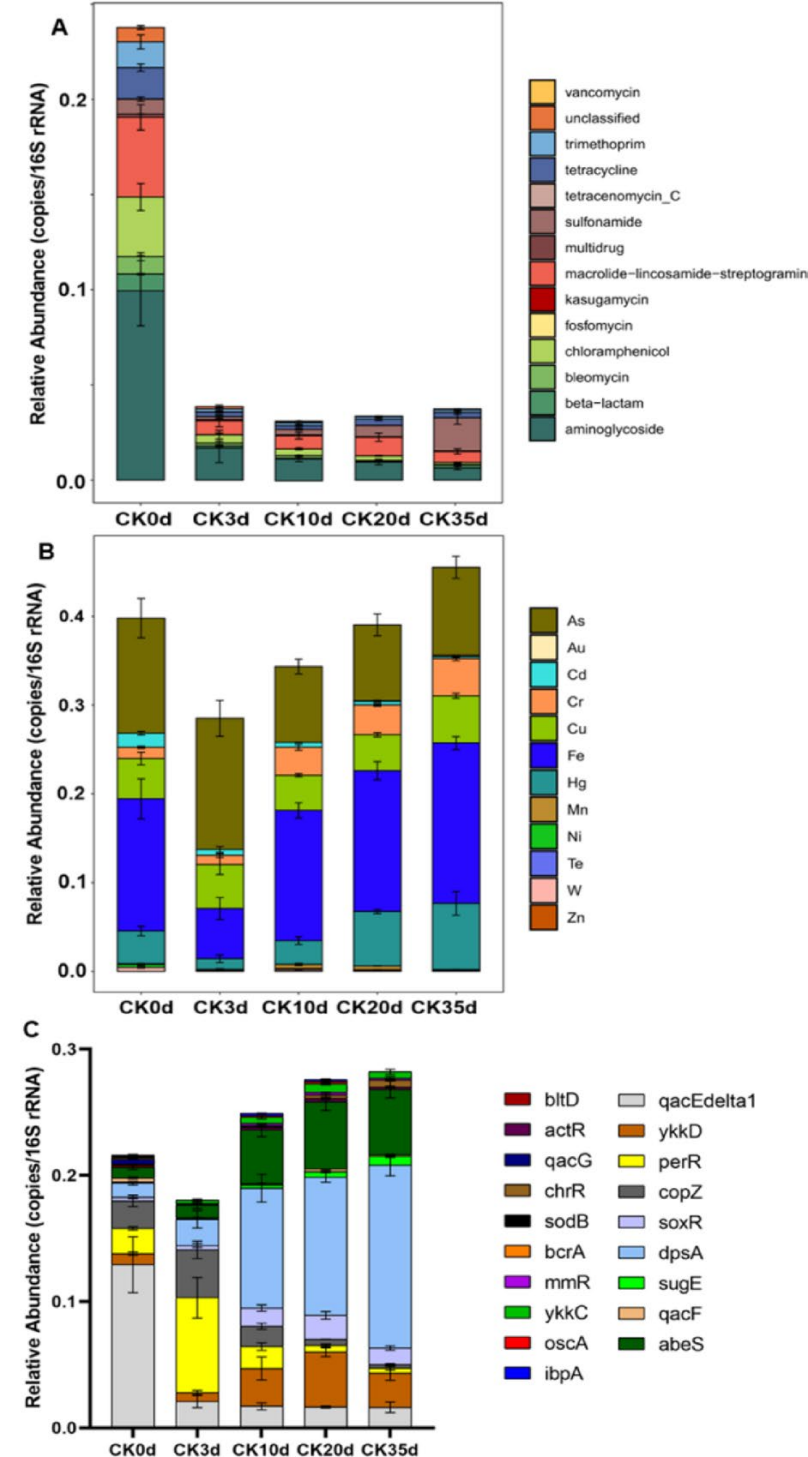
Changes in the relative abundance of ARGs, MRGs, and BRGs during the composting process. (**A**) Variation of relative abundances of ARG types. (**B**) Variation of relative abundances of MRG types. (**C**) Variation of relative abundances of BRG types.

**Fig. 3:**
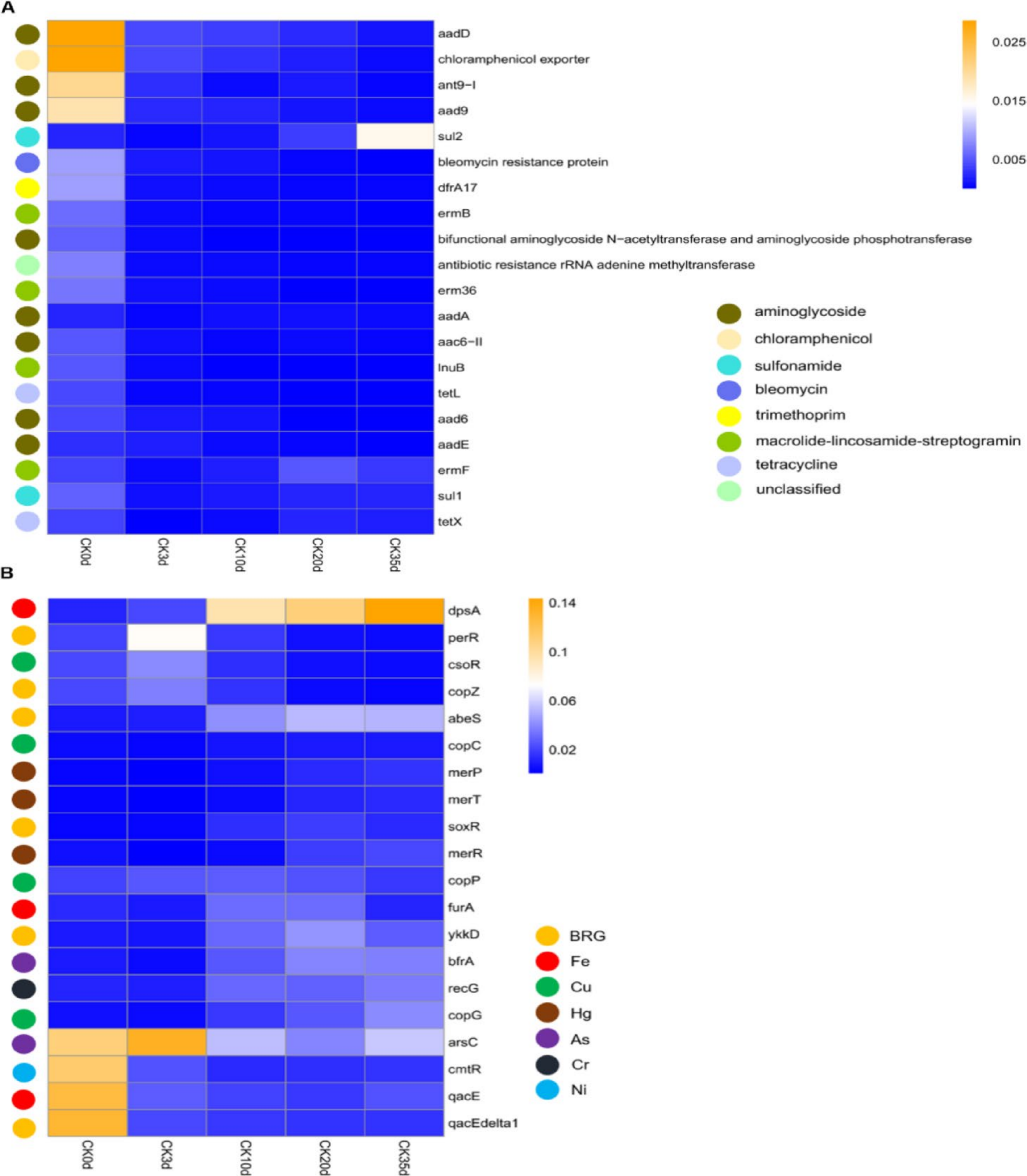
Heatmaps showing changes in the relative abundance of the top 20 ARGs and MRGs during the composting process. (**A**) ARGs Top 20. (**B**) MRGs Top 20.

The results on the dynamics of ARGs are shown in Fig. 2A. There was significant decrease in ARG abundance from 0.237 ± 0.069 copies/16S rRNA on day 0 to 0.038 ± 0.028 copies/16S rRNA on day3 (*P*<0.001), reaching its lowest relative abundance of 0.031 ± 0.006 copies/16S rRNA on day 10. On the other hand, during the later stages of composting, the relative abundance of ARGs exhibited a slight increase, reaching 0.038 ± 0.011copies/16S rRNA at day 35. Nevertheless, this still represented an 86% decrease compared to day 0. Utilizing annotations from the SARG database, the predominant types of detected ARGs during the composting process in this study included aminoglycoside, chloramphenicol, macrolide-lincosamide-streptogram, tetracycline, and trimethoprim. Further selection was conducted on ARG subtypes with relative abundance in the top 20, as illustrated in Fig. 3A. The majority exhibited a decreasing trend in abundance, including aadD, ant9-I, aad9, dfrA17, ermB, erm36, among others. Interestingly, the relative abundance of the sul2 gene within the sulfonamide class showed an increasing trend, which contrasts with the behavior of other types of ARGs.

#### MRGs and BRGs

Some genes were found to exhibit resistance to both heavy metals and biocides, Throughout the composting process, the dynamics of MRGs and BRGs were tracked by annotating sequences against the BacMet database. The total relative abundance of MRGs exhibited a rapid decline from 0.418 ± 0.12copies/16S rRNA on day 0 to 0.347 ± 0.076 copies/16S rRNA by day 3. This trend was then reversed, with levels rising to 0.428 ± 0.32 copies per 16S rRNA by day 35, with no statistically significant differences (*P* > 0.05) observed in abundance variations among sampling timepoints (Fig. 1). Among all identified MRG species, resistance genes related to Fe, Cu, As, and Ni were the predominant types (Fig. 2B).

Annotations from the BacMet database also revealed patterns in the abundance of BRGs, which largely different with the fluctuations observed in ARGs and MRGs. The abundance of BRGs exhibited a progressive increase throughout the composting process. The total BRGs abundance rose from 0.033 ± 0.005 (initial phase, day 0) to 0.15 ± 0.013(mature phase, day 35), with highly statistically significant differences (*P* < 0.01), consistent with previous findings on ARG/BRG dynamics during composting^32,33^. The primary subtypes BRGs identified in compost samples include perR (Hydrogen Peroxide), and abeS (Quaternary Ammonium) (Fig. 3B).

### The co-occurrence of antibiotic, metal, and biocide resistance genes

**Fig. 4:**
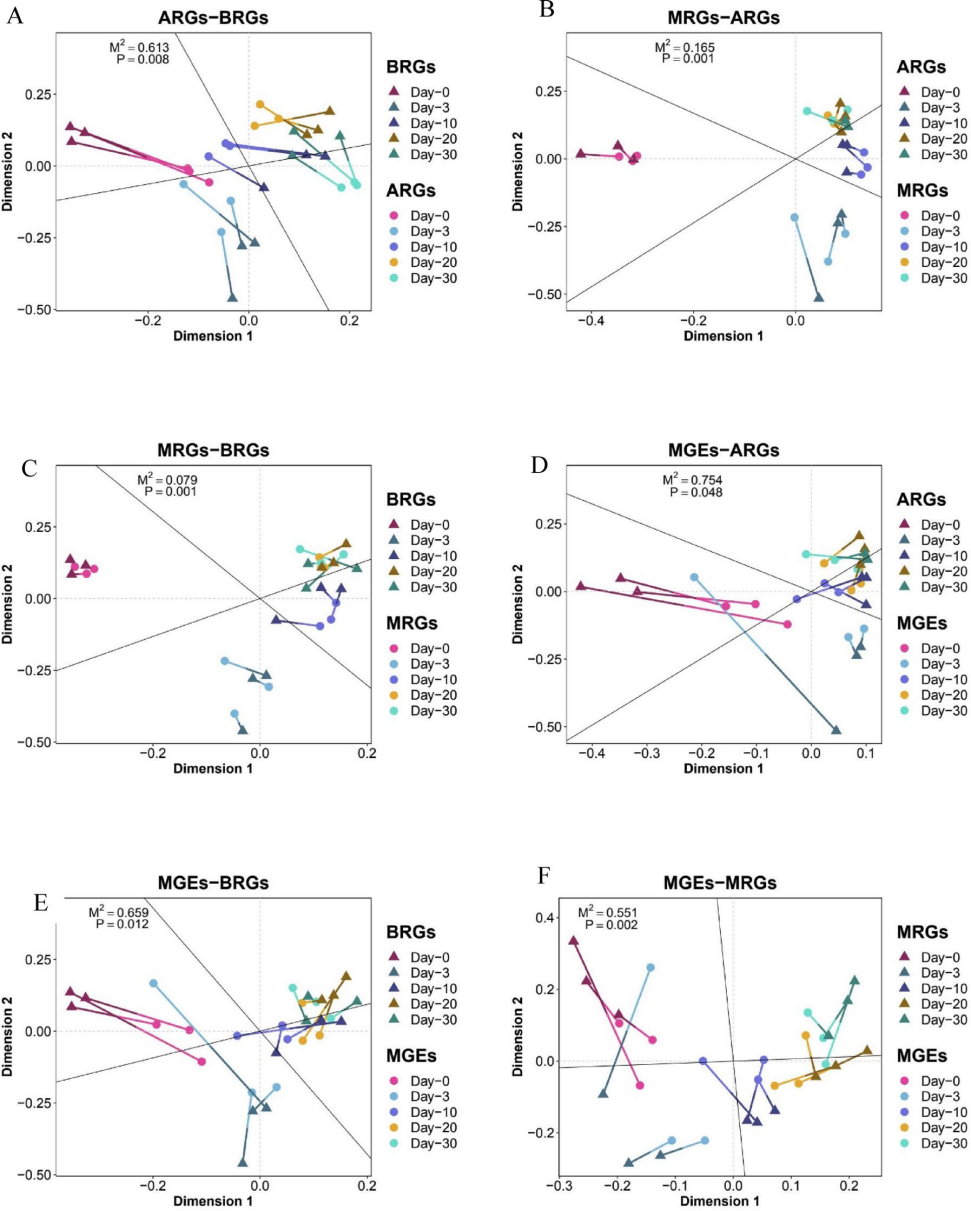
Procrustes analysis of the correlation between (**A**) ARGs on BRGs, (**B**) MRGs on ARGs, (**C**) MRGs on BRGs, (**D**) MGEs on ARGs, (**E**) MGEs on BRGs, (**F**) MGEs on MRGs.

**Fig. 5:**
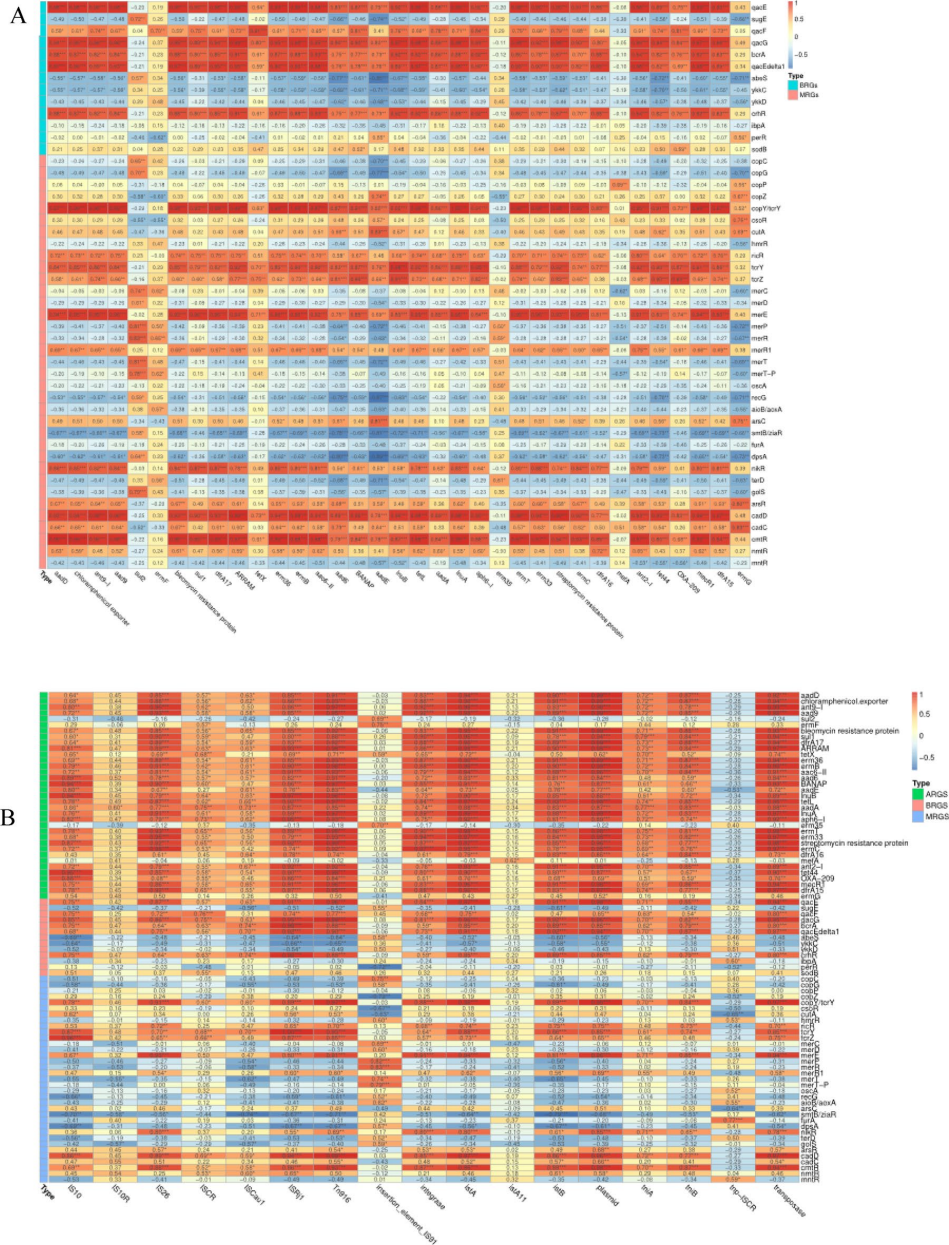
Heatmap of (**A**) BRGs and MRGs in ARGs, (**B**) BRGs/MRGs/ARGs in MGEs.

Procrustes analysis revealed that ARG profiles were significantly correlated with both BRGs (M² = 0.613, *P* < 0.008) and MRGs (M² = 0.165, *P* < 0.001) (Fig. 4a, b). Correlation heatmap analysis further showed strong positive associations (*r* ≥ 0.8, *P* < 0.001) between multiple dominant ARG subtypes and key BRG (e.g., qacE, qacG, bcrA) and MRG (e.g., copY/tcrY, merE) subtypes (Fig. 5A). Although both positive and negative correlations were observed, positive relationships predominated across gene categories.

MGEs were also significantly associated with ARGs (M² = 0.695, *P* = 0.024) and showed a highly significant correlation with MRGs (M² = 0.601, *P* < 0.001). Correlation analysis indicated that major MGEs, including insertion sequences, plasmids, and transposons, were strongly and positively correlated with multiple ARGs, BRGs, and MRGs (*r* ≥ 0.64, *P* < 0.01), suggesting a potential role of MGEs in the co-occurrence and dissemination of resistance genes (Fig. 5B).

### Microbial community dynamics

**Fig. 6:**
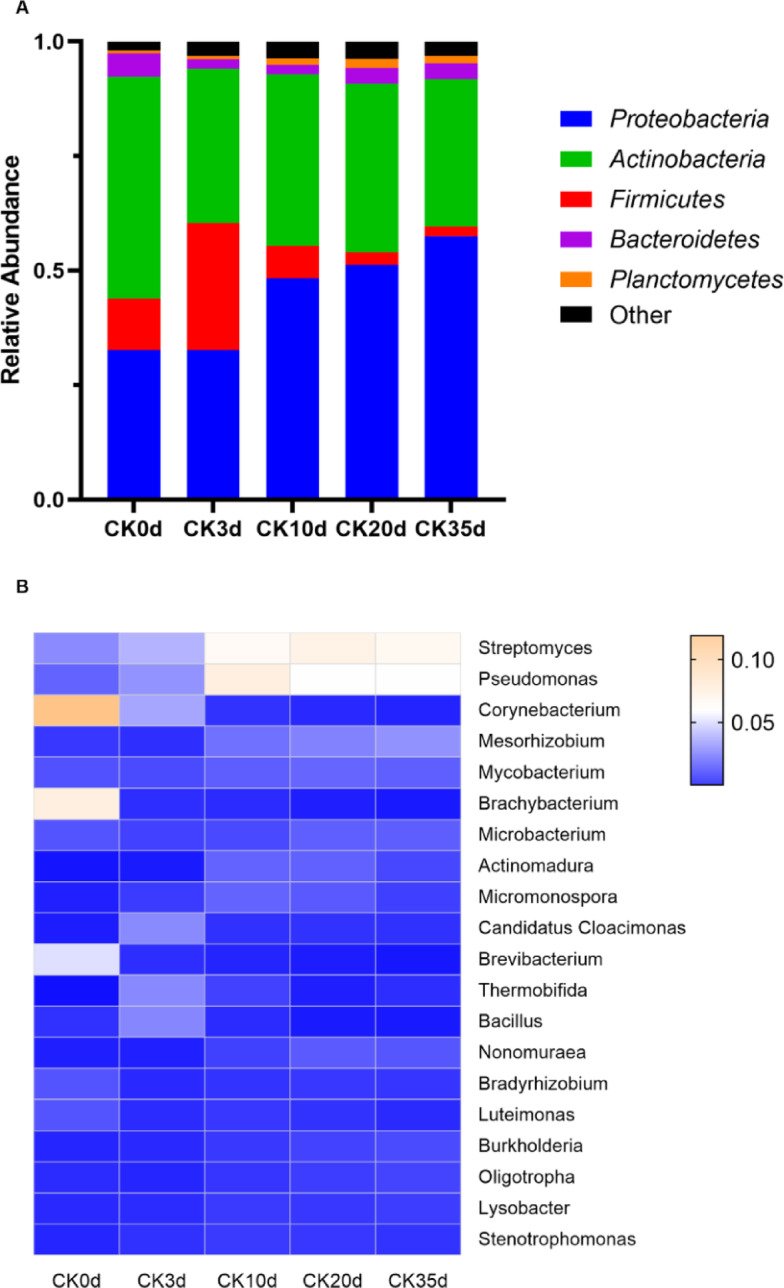
Changes in microbial community composition during the composting process. (**A**) phylum level. (**B**) genus level.

High-throughput sequencing of 16S rRNA genes revealed pronounced shifts in microbial community composition at both the phylum and genus levels during the composting process (Fig. 6A, B). A total of 57 bacterial phyla were detected, with *Proteobacteria*, *Actinobacteria*, Firmicutes, Bacteroidetes, and Planctomycetes collectively accounting for more than 95% of the total community. These dominant phyla are commonly associated with lignocellulose degradation in composting systems. At the phylum level, a clear succession pattern was observed. *Actinobacteria* declined markedly over time, while *Proteobacteria* increased continuously and became the dominant phylum in the later stages. Firmicutes showed a transient increase during the early thermophilic phase, followed by a rapid decrease thereafter.

At the genus level, several *Actinobacteria*-associated genera (e.g., *Corynebacterium*, *Brachybacterium*, and *Brevibacterium*) decreased sharply, consistent with their roles in early, mesophilic composting stages and the depletion of readily degradable substrates. Genera affiliated with Firmicutes (e.g., *Staphylococcus*, *Bacillus*, and *Hungateiclostridium*) exhibited short-lived proliferation during the early phase before declining. In contrast, *Proteobacteria* genera displayed sustained or increasing abundance in the middle and late stages, including *Pseudomonas*, *Mesorhizobium*, and *Castellaniella*, reflecting functional versatility and involvement in nitrogen cycling and degradation of recalcitrant organic compounds.

At the species level, thermophilic taxa served as indicators of composting phases. *Thermobifida fusca* and *Symbiobacterium thermophilum* showed pronounced increases during day 3, confirming the peak thermophilic stage and highlighting their roles in cellulose degradation and high-temperature microbial activity. Their subsequent decline coincided with substrate depletion and temperature reduction, marking the transition toward compost maturation.

### Microbial community shifts on resistance gene Co-selection

**Fig. 7:**
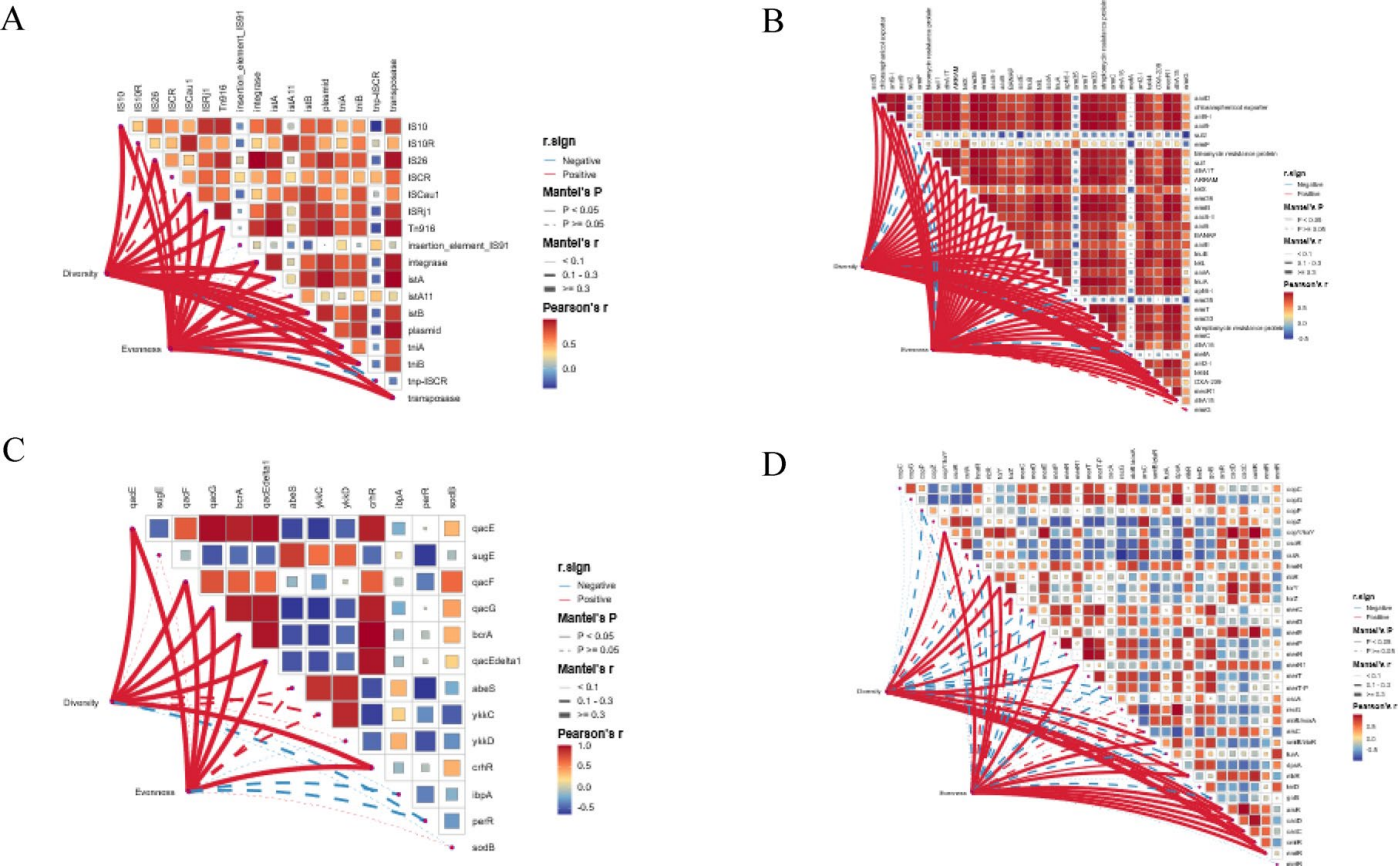
The response between bacterial community characteristics and genes by Mantel test. (**A**) MGEs; (**B**) ARGs; (**C**) BRGs; (D) MRGs. The heatmap shows Pearson’s correlation coefficients between resistance gene profiles (ARGs, BRGs, MRGs) and dominant microbial taxa across composting stages. Warm colors (red) indicate positive correlations, whereas cool colors (blue) indicate negative correlations; color intensity is proportional to the strength of the correlation (Pearson’s r). Only statistically significant correlations are displayed (*P* < 0.05). The curved connecting lines illustrate significant correlations between the two matrices: red lines denote significant positive correlations (*r* > 0), and blue lines denote significant negative correlations (*r* < 0). Line thickness reflects correlation strength, with thicker lines representing higher absolute r values.

The relationships between microbial community characteristics and resistance genes during composting were further examined using Mantel tests (Fig. 7). Overall, microbial diversity and evenness showed strong and widespread positive correlations with most ARGs, BRGs, and MGEs. Among these, MGEs exhibited the strongest associations, underscoring their central role in shaping resistance gene dynamics. Most detected MGEs were significantly and positively correlated with microbial diversity and evenness (*r* > 0.7, *P* < 0.05), with several showing highly significant correlations. Similarly, the majority of dominant ARGs and several BRGs displayed strong positive correlations with community diversity metrics. These consistent patterns suggest that horizontal gene transfer mediated by MGEs is a key mechanism driving the co-occurrence and dissemination of ARGs and BRGs during composting (Fig. 7A–C). In contrast, MRGs exhibited weaker and less consistent relationships with microbial diversity. Only a small subset of MRGs showed significant positive correlations, while several metal- and mercury-resistance genes displayed weak or negative associations. This divergence indicates that MRG dynamics may be regulated by mechanisms distinct from those governing ARGs, BRGs, and MGEs (Fig. 7D).

### Correlation analysis of pathogen changes

**Fig. 8:**
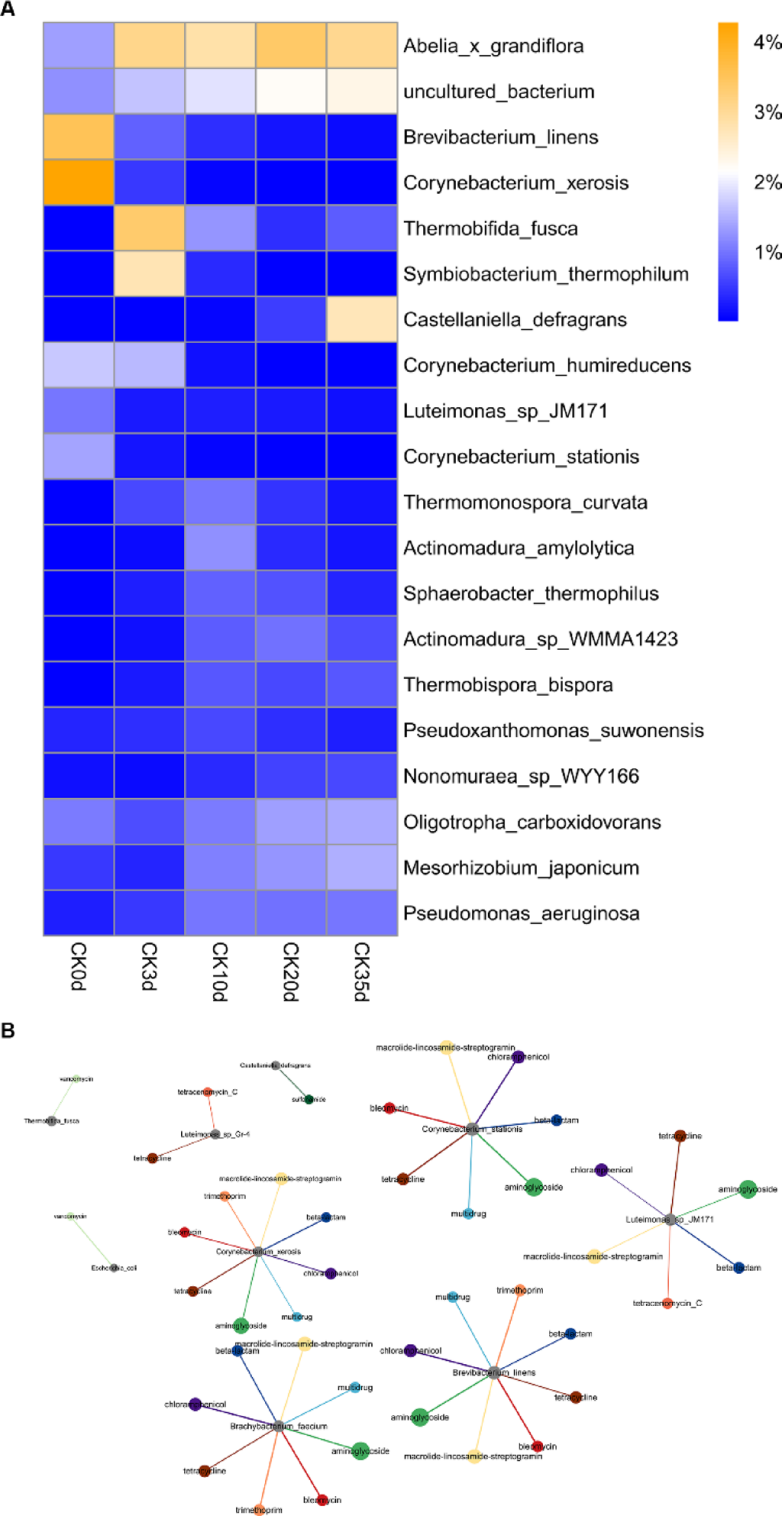
Changes in the relative abundance of pathogenic microbes and their correlation with ARGs. (**A**) Relative abundance of pathogenic microbes. (**B**) Correlation network between pathogenic microbes and ARGs.

As illustrated in Fig. 8A, the initial compost samples showed high concentrations of *Brevibacterium linens* and *Corynebacterium xerosis*, which gradually decreased over time. In contrast, the abundance of *Thermobifida fusca* and *Symbiobacterium thermophilum* increased noticeably three days into the heating period. Additionally, the trend continued with species such as *Abelia grandiflora* and *Castellanella defragrans*, whose abundance gradually increased from day 0 to day 35, indicating their thermotolerance or adaptation to the composting environment.

There was a correlation between various pathogenic microorganisms and ARGs, including *Brevibacterium linens*, *Corynebacterium xerosis*, *Corynebacterium stationis*, *Brachybacterium faecium*, and *Luteimonas sp_JM171*. Among them, there is a correlation with aminoglycoside, macrolide lincosamide streptogram, tetracycline, bleomycin, beta-lactam, and multidrug in ARGs, with the strongest correlation observed with aminoglycoside (Fig. 8B).

**Fig. 9:**
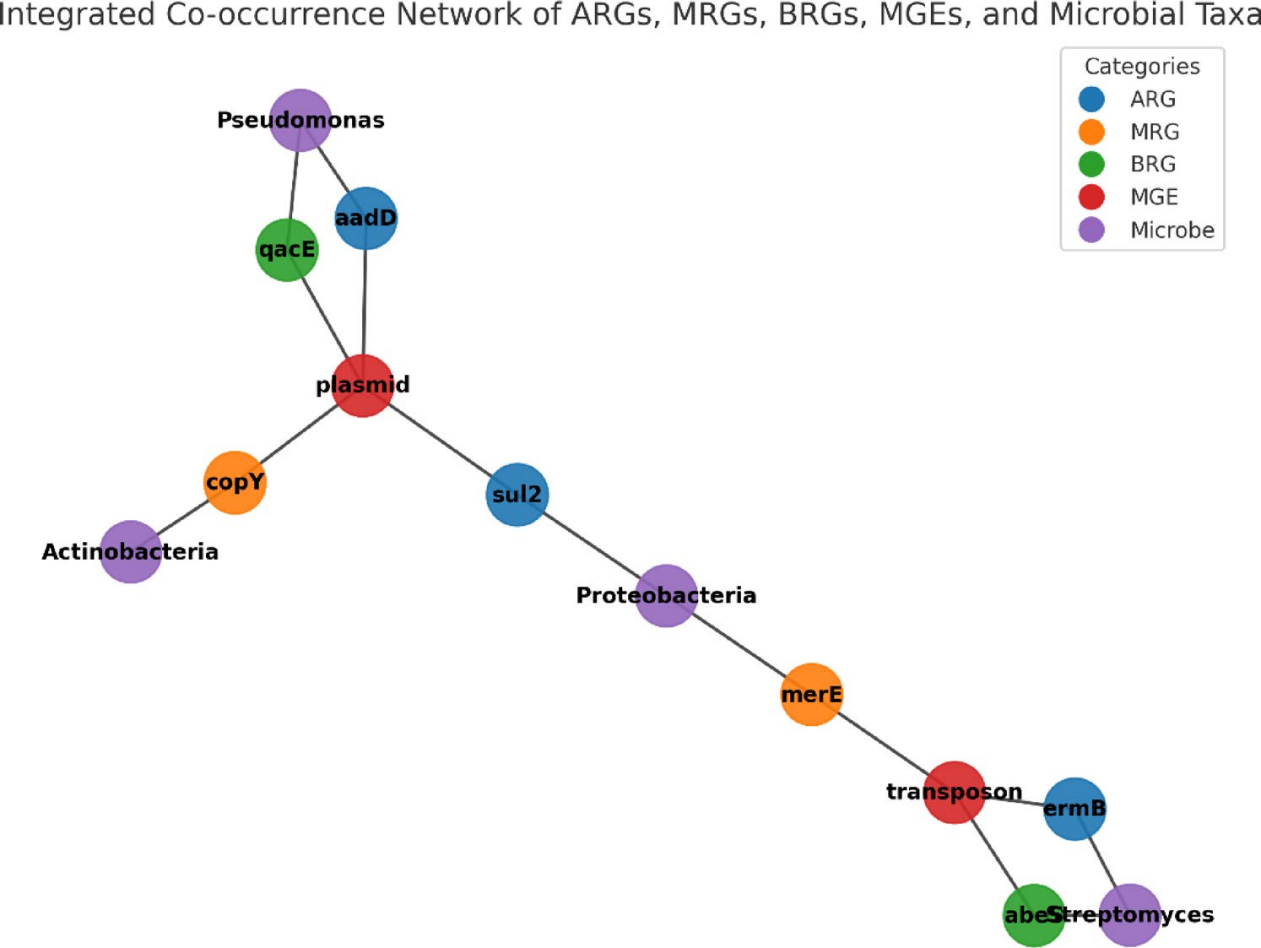
Co-occurrence network linking ARGs, MRGs, BRGs, MGEs, and microbial taxa during dairy manure composting.

The co-occurrence network in the relative abundance of ARGs, MRGs, and BRGs (Fig. 9) revealed that ARGs, MRGs, BRGs, microbial taxa, and MGEs were closely interconnected during the composting process. ARG subtypes such as aadD, ermB, and sul2 were strongly associated with microbial groups including *Pseudomonas*, *Streptomyces*, and members of *Proteobacteria*, reflecting the influence of microbial succession on ARG persistence. MRGs (copY, merE) and BRGs (qacE, abeS) were not only linked to microbial taxa but also showed significant correlations with MGEs, highlighting the role of co-selection in shaping resistance gene dynamics. MGEs (plasmids and transposons) occupied central positions in the network, indicating their importance as hubs for horizontal gene transfer and the potential co-propagation of multiple resistance determinants. These results suggest that the interplay among microbial community shifts, MGEs, and different resistance determinants drives the observed variation trends of ARGs, MRGs, and BRGs during composting.

## Discussion

### Temperature-driven dynamics of ARGs during composting

This study demonstrates that composting temperature is a primary driver shaping antibiotic resistance gene (ARG) dynamics. The rapid decline in ARG abundance during the early, high-temperature phase aligns with previous reports showing that thermophilic conditions can effectively suppress ARG persistence by reducing host viability and degrading extracellular DNA^34–37^. The subsequent partial rebound of ARG abundance during later stages suggests that temperature alone is insufficient for complete ARG elimination, as microbial recolonization and regrowth may occur once favorable conditions return^33,38^. These findings reinforce the view that composting acts as a mitigation, rather than eradication, strategy for ARGs.

### Differential behaviors of ARGs, MRGs, and BRGs

Distinct temporal patterns were observed among ARGs, metal resistance genes (MRGs), and biocide resistance genes (BRGs), indicating that these resistance categories respond differently to composting conditions. While most ARG subtypes declined overall, certain genes such as sul2 persisted or increased, suggesting selective advantages under composting conditions, potentially linked to residual substrates or co-selection pressures^39,40^. In contrast, MRGs generally exhibited increasing trends, consistent with earlier studies reporting enrichment of metal resistance during composting due to stable or accumulating metal residues^37,41^. BRGs showed patterns distinct from both ARGs and MRGs, with some increasing steadily, indicating selective survival of biocide-tolerant populations^32,42^. Together, these results confirm that resistance gene categories are governed by different selective forces during composting.

### Co-occurrence, co-selection, and resistance gene interactions

The observed inconsistency among ARG, MRG, and BRG trends highlights the complexity of resistance gene interactions. Resistance genes rarely act independently; instead, they form interconnected networks shaped by co-selection, shared regulatory mechanisms, and environmental stressors^24,38^. Procrustes analysis revealed strong correlations between ARGs and both MRGs and BRGs, suggesting that fluctuations in metal and biocide resistance may indirectly influence ARG dynamics. These findings are consistent with previous studies demonstrating widespread co-occurrence of ARGs with MRGs and BRGs in manure and compost systems^41,43,44^.

### Role of MGEs and horizontal gene transfer

Mobile genetic elements (MGEs) emerged as central drivers linking resistance genes and microbial communities. Significant correlations between MGEs and both ARGs and MRGs underscore the importance of horizontal gene transfer (HGT) in shaping resistome structure during composting^45,46^. Plasmids, transposons, and integrons likely facilitate rapid dissemination of resistance determinants across diverse microbial hosts, particularly in environments with high microbial diversity and frequent cell-to-cell contact^47,48^. The lack of a significant association between MGEs and BRGs suggests that BRG dynamics may be more strongly influenced by host competition or ecological filtering rather than direct mobilization.

### Microbial succession and resistome dynamics

Microbial community succession played a critical role in shaping resistance gene trajectories. The decline of *Actinobacteria* during the warming phase paralleled reductions in ARG abundance, whereas the transient increase of Firmicutes during thermophilic conditions reflected their tolerance to heat and stress via sporulation^49,50^. Dominant genera such as *Streptomyces* and *Pseudomonas*, belonging to *Actinobacteria* and *Proteobacteria*, respectively, are well-known reservoirs of resistance genes and exhibited strong associations with ARGs throughout composting^51,52^. Mantel test results further revealed that microbial diversity and evenness were positively correlated with MGEs, ARGs, and BRGs, indicating that a diverse microbial background enhances opportunities for HGT and resistance gene maintenance.

### Integrated network perspective and global implications

Network analysis highlighted the tightly interconnected relationships among microbial taxa, ARGs, MRGs, BRGs, and MGEs. Central positioning of plasmids and transposons confirmed their role as key hubs facilitating resistance gene exchange, consistent with prior findings in manure- and soil-based systems^53–55^. These interactions demonstrate that resistomes must be considered as integrated ecological systems shaped by microbial succession, co-selection pressures, and gene mobility. International studies from China, Europe, and beyond similarly report that composting cannot fully decouple ARGs from MRGs and BRGs, particularly in systems influenced by heavy metals and biocides^56–59^. Collectively, these findings emphasize that effective antimicrobial resistance mitigation in livestock waste management requires integrative strategies that account for microbial ecology, MGEs, and multiple co-selective pressures rather than targeting ARGs alone^60^.

### Limitation of the study

The composting experiment was performed under controlled, small-scale conditions, which may not fully represent large-scale or natural composting environments. Compost materials were obtained from limited local sources, potentially restricting the generalizability of results. The selected sampling intervals may have missed rapid microbial and gene shifts during critical composting phases. Additionally, DNA extraction and database annotation biases could have influenced microbial and resistance gene profiles, and functional validation of the detected genes was not conducted. Important environmental parameters such as oxygen gradients and microbial activity were not continuously monitored, and limited replication may have reduced statistical robustness. Moreover, the focus on antibiotic and metal resistance genes excluded other gene types like virulence factors or mobile genetic elements. The 35-day duration may not capture long-term compost stabilization, and the environmental risks of resistance gene persistence in the final compost were not evaluated.

## Conclusion

- This study underscores the complex and dynamic nature of resistance gene behavior—specifically antibiotic resistance genes (ARGs), metal resistance genes (MRGs), and biocide resistance genes (BRGs), during the dairy manure composting process. A key observation was the initial rapid decline in ARG abundance, closely associated with the thermophilic phase, indicating that high composting temperatures can effectively suppress many ARG subtypes. However, the resurgence of certain ARGs in the later stages, along with the increase in MRGs and BRGs, reveals that composting alone may not ensure the complete elimination of resistance determinants.
- The variable trends among ARGs, MRGs, and BRGs suggest that resistance genes respond differently to composting conditions due to unique microbial hosts, selective environmental pressures, and varying mechanisms of horizontal gene transfer (HGT). While MRGs showed consistent increases, possibly due to heavy metal enrichment and host microbial survival, BRGs exhibited a progressive rise, indicating resilience and possible selection under composting conditions.
- Importantly, the co-occurrence and significant correlations among ARGs, MRGs, BRGs, and mobile genetic elements (MGEs) highlight the potential for co-transfer and persistence of resistance traits via plasmids, integrons, and transposons. MGEs were strongly associated with ARGs and MRGs but not with BRGs, suggesting differing mechanisms of gene propagation. Co-selection mechanisms—such as cross-resistance and co-regulation—appear to play a critical role in sustaining ARGs in the presence of biocides and heavy metals.
- Microbial community dynamics were closely tied to resistance gene patterns. The dominance of *Proteobacteria* and *Actinobacteria*, particularly *Pseudomonas* and *Streptomyces*, paralleled the abundance of aminoglycoside and other ARGs, reflecting the ecological niche and metabolic capacities of these genera. The shift from *Actinobacteria* to *Proteobacteria* dominance further suggests that compost microbial succession may influence resistance gene retention.

## Recommendations

From the study findings, it is recommended that there is need;

- To maintain high thermophilic temperatures for extended periods and prolong composting duration to maximize ARG reduction and prevent gene resurgence in later stages.
- To limit the use of antibiotics, heavy metals, and biocides in livestock practices; pre-treat manure to reduce ARG, MRG, and BRG loads before composting.
- To routinely track ARGs, MRGs, BRGs, and MGEs using molecular tools to assess compost safety and manage the risk of horizontal gene transfer.
- To encourage the growth of non-resistant, competitive microbes through microbial inoculants or process adjustments to suppress resistance gene carriers.

## Data Availability

The datasets used and/or analysed during the current study are available from the corresponding author on reasonable request.
